# Mitochondria: A Therapeutic Target for Parkinson’s Disease?

**DOI:** 10.3390/ijms160920704

**Published:** 2015-09-01

**Authors:** Yu Luo, Alan Hoffer, Barry Hoffer, Xin Qi

**Affiliations:** 1 Department of Neurological Surgery, Case Western Reserve University, Cleveland, OH 44106, USA; E-Mails: Alan.Hoffer@uhhospitals.org (A.H.); bhoffer@intra.nida.nih.gov (B.H.); 2 Department of Physiology, Case Western Reserve University, Cleveland, OH 44106, USA

**Keywords:** Parkinson’s disease, mitochondrial dysfunction, mitochondrial dynamics

## Abstract

Parkinson’s disease (PD) is one of the most common neurodegenerative disorders. The exact causes of neuronal damage are unknown, but mounting evidence indicates that mitochondrial-mediated pathways contribute to the underlying mechanisms of dopaminergic neuronal cell death both in PD patients and in PD animal models. Mitochondria are organized in a highly dynamic tubular network that is continuously reshaped by opposing processes of fusion and fission. Defects in either fusion or fission, leading to mitochondrial fragmentation, limit mitochondrial motility, decrease energy production and increase oxidative stress, thereby promoting cell dysfunction and death. Thus, the regulation of mitochondrial dynamics processes, such as fusion, fission and mitophagy, represents important mechanisms controlling neuronal cell fate. In this review, we summarize some of the recent evidence supporting that impairment of mitochondrial dynamics, mitophagy and mitochondrial import occurs in cellular and animal PD models and disruption of these processes is a contributing mechanism to cell death in dopaminergic neurons. We also summarize mitochondria-targeting therapeutics in models of PD, proposing that modulation of mitochondrial impairment might be beneficial for drug development toward treatment of PD.

## 1. Introduction

Parkinson’s disease (PD) is the second most common neurodegenerative disorder after Alzheimer’s disease, affecting over 1% of the population older than 60 years of age. Clinically, it is diagnosed primarily based on motor abnormalities including bradykinesia, resting tremor, and cogwheel rigidity [1]. A key characteristic of pathology in PD is the degeneration of the nigrostriatal (NS) dopaminergic pathway which is one of the most important dopamine (DA) pathways in the brain and contains about 80% of the total brain DA. Despite a large number of studies on the pathogenesis of PD, there is still inconclusive evidence about why dopaminergic neurons are selectively degenerated. Currently, there is no effective restorative treatment available for PD, only symptomatic treatment is available.

Among a number of proposed mechanisms involved in PD pathogenesis, mitochondrial dysfunction has been repeatedly implicated as the cause of the death of DA neurons in PD [2,3,4,5]. Mitochondria are critical for many cellular functions, such as intermediary metabolism [6,7], redox signaling [8], calcium homeostasis [9,10,11], cell proliferation [12,13], development [14,15] and cell death [16,17,18]. Mitochondrial dysfunction is mainly characterized by the generation of reactive oxygen species (ROS), a defect in mitochondrial electron transport complex enzyme activities, ATP depletion, caspase 3 release and depletion of mitochondrial DNA. In this review, we summarize evidence on the critical involvement of mitochondria in both genetic mutation and environmental toxin-induced PD. We propose a causal role for mitochondrial dysfunction in the development of PD, because (1) neurotoxins causing parkinsonism, such as 1-methyl-4-phenyl-1,2,3,6-tetrahydropyridine (MPTP), rotenone, paraquat, induce dopaminergic neuronal death through direct inhibition of mitochondrial complex I activity; (2) mutant proteins from PD-related genes associate with mitochondria where they elicit diverse mitochondrial dysregulation and subsequently cause neuronal degeneration; (3) therapeutic agents that target mitochondrial protein or inhibit mitochondrial damage can reduce neuropathological phenotypes of PD in animal models and cells from PD patients.

## 2. Mitochondrial Dysfunction in Parkinson’s Disease

Aberrant mitochondrial function is one of the major cytopathologies in PD and has been widely accepted as a central pathogenic mechanism underlying PD pathogenesis. Chronic systemic administration of rotenone, a specific complex I inhibitor and a pesticide, results in neuropathologic and behavioral changes in rats that are similar to human PD [19,20]. 1-methyl-4-phenyl-1,2,3,6-tetrahydropyridine (MPTP), a meperidine analog found to cause parkinsonism in humans, exerts its toxic effects through metabolism to 1-methyl-4-phenylpyridinium (MPP^+^), another complex I inhibitor [19,21]. These compounds have long been used in animal models of PD. Furthermore a number of familial forms of PD are associated with mutations in genes encoding both mitochondrially targeted proteins and proteins involved in mitochondrial function and/or oxidative stress responses, including mutations in PINK-1, DJ-1, parkin, and leucine-rich repeat kinase 2 (LRRK2) [22]. Interestingly, one study reported that the size of mitochondria in dopaminergic neurons in the substantia nigra (SN; susceptible to PD degeneration) is smaller than in neighboring non-dopaminergic neurons or in dopaminergic neurons of the ventral tegmental area which is more resistant in PD, suggesting a basis for the increased vulnerability of SN neurons to subtle changes in mitochondrial maintenance and function [23]. In addition, increased oxidative stress due to mitochondrial compromise in PD model animals has been proposed to contribute to the degeneration of dopaminergic neurons [23].

Consistent with the evidence from basic science, clinical studies also showed that mitochondrial damage plays a predominant role in the development of PD in patients. Mild deficiency in mitochondrial respiratory electron transport chain NADH dehydrogenase (Complex I) activity has been reported in the substantia nigra [24] as well as platelets [25,26] and lymphocytes [27,28] in PD patients, suggesting a systemic inhibition of complex I activity in PD patients. Mitochondrial dysfunction could lead to increased oxidative stress. Indeed oxidative damage to lipids, proteins and DNA has been detected in brain tissue from PD patients [29,30]. A recent proteomic analysis of mitochondria-enriched fractions from post-mortem PD substantia nigra revealed differential expression of multiple mitochondrial proteins in PD brains as compared with control brains [23]. In further support for a “mitochondrial genetics” hypothesis for PD pathophysiology, Bender *et al.* [31] reported higher levels of mitochondrial DNA deletions in nigral neurons from PD patients. Moreover, both Bender *et al.* [31] and Kraytsberg *et al.* [32] reported higher levels of mitochondrial DNA deletions in nigral neurons of aged humans with sharp elevations starting shortly before age 70. This correlates with the known risk factor of age in PD. It is possible that there is an accumulation of mitochondrial dysfunction and of reactive oxygen species (ROS) damage during aging which needs to reach a critical threshold for cellular dysfunction and degeneration to be observed. How a systemic dysregulation in mitochondrial function or oxidative damage leads to tissue or cell type specific vulnerability still remains to be elucidated.

Taken together, although studies over many years on PD indicate an important role of mitochondria in PD-associated pathology, the process by which the mitochondria become dysfunctional in PD and whether correction of mitochondrial defects could provide neuroprotection in PD remain to be determined.

## 3. Environmental Toxins that Influence Mitochondrial Function

Some toxins used to model DA loss in PD, such as MPTP and rotenone, impair respiratory chain function by inhibiting complex I [33,34,35,36]. These complex I inhibitors replicate some of the key motor features of PD and lead to DA neuronal loss. Intravenous injection of the compound MPTP by drug addicts caused a condition that closely resembles the anatomic and clinical features of PD [37,38]. Multiple models have been developed in the laboratory in which the chronic infusion of the pesticide rotenone or combination of herbicides and pesticides [39] lead to a pattern of cell death and DA loss similar to that of PD. The precise role of environmental toxins in the cause of PD remains to be defined, but these data support the hypothesis that environmental toxins could introduce mitochondrial dysfunction and lead to parkinsonism in human. Indeed, epidemiological studies showed that the prevalence of sporadic PD is higher among farming communities [40]. Exposure to pesticides or herbicides elicited a three-to fourfold increased risk of developing PD [41]. All of these data suggest an environmental contribution to the etiology of sporadic PD.

## 4. Genetic Factors Associated with PD

In a twin study, Tanner *et al.* showed that for early onset cases, monozygotic concordance was twice that of dizygotic concordance, suggesting that genetic factors are important in the early onset PD [42]. Many examples of familial parkinsonism have also been reported (See review [43]). Mutations of several genes have been linked to familial PD and parkinsonian syndromes [44,45,46].

### 4.1. Mitochondrial DNA Mutations and Deletions in PD

Mitochondrial DNA (mtDNA) encodes 13 subunits of respiratory chain proteins, including seven complex I, one complex III, three complex IV, and two complex V subunits. Until now, there has been no PD-associated genetic mutations in mtDNA reported [47]. However, mtDNA deletions have been observed in individual dopaminergic neurons dissected from postmortem human substantia nigra tissue [31]. In addition, mutations in the gene encoding human mtDNA polymerase subunit γ (POLG) leads to clinical parkinsonism associated with multiple mtDNA deletions. [48,49]. Furthermore, proliferator-activated coactivator-1 α (PGC-1 α), one of the key regulators of mitochondrial biogenesis, was found to be decreased in PD patients through a genome-wide association study (GWAS) [50]. Directed deletion of transcriptional factor A (TFAM) in mouse DA neurons using the Cre-LoxP system, termed as MitoPark mice, causes marked deletion of mtDNA, severe impairment of oxidative phosphorylation and slowly progressive motor deficits in the DA system that mimic human parkinsonism as well as altered response to L-3,4-dihydroxyphenylalanine (L-DOPA) treatment [51,52,53]. The MitoPark mice of PD provided direct evidence that mitochondrial dysfunction in DA neurons can causes PD-related phenotypes. Consistently increased level of mtDNA deletions in the striatum of PD patients have been reported [54] and mtDNA deletions were significantly higher in neurons with impaired cytochrome oxidase activity [31,32]. These findings support a mitochondrial genetic contribution in PD.

### 4.2. Nuclear Gene Mutations Affecting Mitochondrial Function

Many of the PD susceptible genes identified are related to mitochondrial function (Table 1). These genes and their potential contribution to PD have previously been extensively reviewed [43]. PD linked genes that affect mitochondrial function include, but are not limited to, α-synuclein, Parkin, Phosphatase and tensin homologue (PTEN)-induced putative kinase 1 (PINK1), DJ-1 and LRRK2. In this review we will focus on their involvement and effects on dysregulation of mitochondrial dynamics, mitophagy and mitochondrial redox, and mitochondrial protein import.

*Alpha-synuclein*: α-synuclein is aggregation-prone protein which attains an increased propensity to aggregate because of the presence of its hydrophobic non-amyloid β component domain. Missense mutations in the alpha-synuclein gene are associated with autosomal dominant PD [55]. The fibrillar form of α-synucleinis a major component of Lewy bodies and has been demonstrated to trigger neurotoxicity in PD [56,57].

*Parkin*: Parkin is a RING finger containing ubiquitin E3 ligase. It is known to mediate poly-ubiquitination of its substrates for proteasomal degradation. Mutations in the parkin gene cause early onset juvenile autosomal recessive PD, and Parkin mutations are the most common cause of young onset PD. The loss of parkin E3 ligase activity results in accumulation of its substrates leading to neurotoxicity in autosomal recessive PD [58,59].

*PINK1*: Phosphatase and tensin homologue (PTEN)-induced putative kinase 1 (PINK1) is a serine/threonine kinase localized in mitochondria. Mutations in PINK1 are associated with a rare form of autosomal recessive PD. PINK mutations result in the loss of PINK1 function which leads to aberrant phosphorylation of its substrates to cause PD [60,61].

**Table 1:** Animal models of Parkinson’s disease (PD)-related genes affect mitochondrial function.

Animal Models	Genetic Manipulation in Animals	Motor Phenotypes	PD Pathology and Mitochondrial Function	References
Alpha-Synuclein transgenic mice	hA53T alpha-Synuclein in mice; mPrP promoter	Severe leading to paralysis and premature death	Lewy body-like inclusion in older mice; mitochondrial dysfunction; no dopaminergic neuronal loss	[62,63,64]
	hA30P alpha-synuclein in mice; mThy-1 promoter	Severe leading to paralysis	Lewy body-like inclusion; sensorimotor neuronal loss in brain stem	[65]
	Alpha-synuclein overexpression in mice (Thy1 promoter)	Progressive declines in spontaneous and motor activity	No DA neuronal degeneration, mitochondrial dysregulation	[66,67]
	hA53T alpha-synuclein expressing in SN DA neurons of mice	Body weight loss; normal locomotion activity	Progressive DA neuronal loss; aberrant mitochondrial inclusion	[68]
LRRK2 transgenic or knock-in mice	LRRK2 R1441G mice; BAC promoter	Rearing activity decrease in older mice	DA neurite degeneration; Tau phosphorylation increase; no DA neuronal degeneration; mitochondrial dysfunction	[69,70]
	LRRK2 G2019S knock-in mice	Absent	Abnormal mitochondrial morphology; mitochondrial dysfunction; no DA neuronal loss	[71]
Parkin^−/−^ mice	Parkin germline inactivation	Conflicting: either absent or subtle motor movement disturbance	Absent	[72,73]
DJ1^−/−^ mice	DJ1 germline inactivation	Age-dependent declines in locomotor activity	Impaired DA update; no DA neuronal degeneration	[74]
PINK1^−/−^ mice	PINK1 germline inactivation	Age-dependent declines in spontaneous activity	Impaired DA update; no DA neuronal degeneration; mitochondrial abnormalities	[75,76]
Mito-Park mice	DAT riven cre; loxed p TFAM	Begins at 3–4 months; declines in spontaneous and rearing activity	Abnormal mitochondrial aggregates; DA reduction in the striatum; progressive DA neuronal degeneration	[51]
Double-mutant mice	A53T alpha-synuclein overexpression in Parkin^−/−^ mice	Absent	Altered mitochondrial structure and morphology; no DA neuronal loss	[77]

*DJ-1*: DJ-1 belongs to the peptidase C56 family of proteins. Wild-type DJ-1 can serve as a chaperone, protease, regulator of transcription and autophagy through redox regulation. DJ-1 seems to be cytoprotective specifically under conditions related to oxidative stress. Its protective action is the result of the modification of cysteine residues on DJ-1 to cysteine-sulfinic and cysteine-sulfonic acids under oxidative stress [78]. DJ-1 mutations associated with PD are rare and account for 1%–2% of autosomal recessive early-onset PD [79].

*LRRK2*: Leucine-rich repeat kinase 2 (LRRK2) is a protein encoded by the PARK8 locus. It has a conserved serine-threonine kinase mitogen-activated protein kinase kinase kinase (MAPKKK) domain, a member of the Roc (Ras of complex) GTPase family [80,81,82]. To date, there are over 50 variants identified in PD patients. The mutation G2019S (Gly2019 to Ser) that takes place in the MAPKKK domain has been recognized as the most common cause of dominant familial PD and accounts for up to 2% of sporadic PD cases [83]. The G2019S mutant augments the kinase activity of LRRK2, which is associated with increased toxicity in dopaminergic neurons [84].

### 4.3. Mitochondrial Dynamics Impairment in Parkinson’s Disease

Mitochondria are organized in a highly dynamic tubular network that is continuously reshaped by opposing processes of fusion and fission [85]. Mitochondrial fission and fusion were first observed in yeast, and since have been observed in all mammalian cells [86]. A delicate balance is maintained between fusion and fission to ensure the normal function of mitochondria. Specifically the fusion process is important for mitochondrial interactions and communication, and fission facilitates the segregation of mitochondria into daughter cells and enhances mitochondrial renewal and distribution along cytoskeletal tracks. Fusion and fission events enable the proper exchanging and mixing of mitochondrial membranes and contents. This dynamic process controls not only mitochondrial morphology, but also the subcellular location and function of mitochondria. Defects in either fusion or fission limit mitochondrial motility, decrease energy production and increase oxidative stress, thereby promoting cell dysfunction and death [87,88]. The two opposing processes, fusion and fission, are controlled by evolutionarily conserved large GTPases that belong to the dynamin family of proteins. In mammalian cells, mitochondrial fusion is regulated by mitofusin-1 and -2 (MFN1/2) and optic atrophy 1 (OPA1), whereas mitochondrial fission is controlled by the dynamin-1-related protein, (Drp1) [89,90] and its mitochondrial adaptors such as Fis1, Mff and MiD49/51 [91,92,93]. Drp1 is primarily found in the cytosol, but it translocates from the cytosol to the mitochondrial surface in response to various cellular stimuli to regulate mitochondrial morphology [1]. At the mitochondrial surface, Drp1 is thought to wrap around the mitochondria to induce fission using its GTPase activity [94].

In terms of PD, no mutations in typical mitochondria fission and fusion genes have yet been identified in PD patients. However, increasing evidence from both toxin models and genetic mutations in PD animal models supports the hypothesis that mitochondrial dynamic regulation and dysfunction are involved in PD. Evidence from toxin-induced PD models support a role for mitochondrial fission/fusion in the pathogenesis of PD. The Parkinsonian neurotoxins, 6-hydroxy dopamine (6-OHDA), rotenone, and MPP^+^, all induce mitochondrial fragmentation, leading to dopaminergic cell death in neuronal cultures [91,95,96]. Inhibition of pro-fission Drp1 or overexpression of pro-fusion protein mitofusin-1 (Mfn1) using genetic techniques prevents both neurotoxin-induced mitochondrial fission and neuronal cell death [97,98,99]. Loss of Mfn2 or conditional knockout of neuronal Mfn2 in mice has recently been reported to result in age-dependent motor deficits, followed by the loss of dopaminergic terminals in the striatum [100,101], suggesting a role for Mfn2 in parkinsonism. Also, Drp1 is required for synaptic formation [102] and lack of Drp1 leads to an impairment of brain development in mice [103,104]. Recently it has been reported that the Drp1 is critical for targeting mitochondria to the terminal synapses of dopaminergic neurons and deletion of Drp1 gene in dopaminergic neurons rapidly eliminates DA terminals in the caudate-putamen and causes cell bodies in the midbrain to degenerate and lose α-synuclein [105]. Taken together, all these support that molecular machinery which maintains the balance of fusion and fission dynamics in the cells might contribute to the pathogenesis of PD. 

In addition, several genes, whose mutated forms are associated with familial PD, affect mitochondrial dynamics: these include PINK1, Parkin, LRRK2 and DJ-1 [45,94,96,106,107,108,109,110]. Among these genes, the role of PINK1/Parkin pathway in regulation of mitochondrial dynamics seems to be opposite from LRRK2 and DJ-1; mutations in PINK1/Parkin lead to mitochondrial fusion [44,111] whereas mutations in LRRK2 or DJ-1 promote mitochondrial fission [44,112]. Fibroblasts from PD patients carrying PINK1 or Parkin mutations exhibited a more fragmented mitochondrial network, showing mitochondrial dysfunction [113,114]. The mitochondrial network could also be reduced by the depletion of Drp1, or overexpression of OPA1 or Mfn2 [115,116,117,118]. Deficiency in DJ-1 in cell lines, cultured neurons and lymphoblasts derived from DJ-1-deficient patients displayed aberrant mitochondrial morphology [119]. Further, in double-mutant mice in which alpha-synuclein mutant is overexpressed and parkin is ablated, severe genotype-, age- and region-dependent mitochondrial morphological alterations were found in neuronal somata. The number of structurally altered mitochondria was significantly increased in the SN of these double-mutants mice [77]. These studies further support the involvement of vulnerable genes in mitochondrial dynamics regulation in PD.

Wild-type LRRK2 interacts and colocalizes with several key regulators of mitochondrial fusion/fission, suggesting that it might have multiple regulatory roles [120]. Furthermore, mutant *LRRK2* G2019S, the most common mutation in the population of familial PD patients, has been recently demonstrated to interact with Drp1 and to promote mitochondrial fragmentation, leading to mitochondrial dysfunction and neuronal abnormalities [121,122]. This fragmentation can be reduced by expression of the dominant-negative Drp1K38A or overexpression of the fusion protein Mfn2 [121,122]. iPS cells derived from PD patients carrying the G2019S mutation show excessive mitochondrial fission, aberrant autophagy and neuronal damage in DA neurons differentiated *in vitro* [123]. More importantly, treatment with P110, a selective peptide inhibitor of Drp1 recently developed in Qi’s group [124,125], reduced mitochondrial fragmentation and damage, and corrected excessive autophagy. In this study it was also shown that G2019S mutated LRRK2 protein primarily phosphorylates Drp1 at T595 resulting in aberrant mitochondrial fragmentation [123].

Together, these findings suggest that impairment of mitochondrial dynamics might contribute to the pathogenesis and progression of PD.

## 5. Mitophagy/Autophagy Impairment in Parkinson’s Disease

Autophagy is a process of cellular degradation in which cargos are degraded by autophagosomes fused with lysosomes [126]. In addition to non-selective cargos, autophagosomes can degrade protein aggregates or damaged mitochondria. Mitochondria-associated autophagy (mitophagy) is regulated by autophagy receptors that preferentially bind ubiquitylated mitochondria and subsequently recruit the autophagosome protein light chain 3 (LC3) through their LC3-interaction region (LIR) motif [127]. LC3 in mammals, also known as Atg8 in yeast, plays crucial roles in both autophagosome membrane biogenesis and cargo recognition [128,129]. In yeast, Atg32 functions as a receptor on mitochondria to initiate mitophagy through its interaction with Atg8 [130,131]. In mammals, FUNDC1 [132], p62 [133], BNIP3 [134], and AMBRA1 [135], have been recognized as receptors for mitophagy, all of which bind to LC3 via the LIR motif [136]. The implication of these receptors in mitophagy regulation, however, seems to be dependent of experimental conditions. Whether and how these receptors cooperatively regulate mitophagy remains to be determined. 

A number of lines of evidence suggest a critical role for defective autophagy/mitophagy in neurodegeneration in PD. Cultured cells exposed to parkinsonian neurotoxins such as MPP^+^, rotenone, or 6-OHDA showed an increased number of autophagosomes and associated neuronal cell death [125,137,138]. In recent years, PINK1-depenent activation of parkin has been recognized as a major pathway of mitophagy [139]. In mammalian cells, parkin is recruited to depolarized mitochondria, which are subsequently eliminated by autophagy. Such parkin recruitment to mitochondria depends on PINK1 accumulation on mitochondria and therefore PINK1 is a key molecule in the signal transduction of mitophagy [140]. The failure of PINK1/parkin-mediated mitophagic process leads to accumulation of damaged mitochondria, which results in an increase in ROS and cell death [141,142]. Because these studies were all conducted in cells with overexpression of PINK1 and Parkin and because Parkin at endogenous levels fail to mediate mitophagy in PD patient cells [143], the matter of whether these proteins, at the endogenous levels, cooperatively regulate mitophagy remains to be validated. Knock-down of DJ-1, another PD-related gene, resulted in decreased mitochondrial membrane potential, increased reactive oxygen species, excessive mitochondrial fragmentation and impaired autophagy [74,119,144]. Interestingly, overexpression of PINK1 and parkin can rescue mitochondrial fragmentation and dysfunction induced by the depletion of DJ-1 [145,146]. Given that wild-type and mutant DJ-1 can interact with PINK1 [147] and that Parkin, as an E3 ligase on mitochondria, catalyzes the ubiquitination of DJ-1 [148], PINK1, Parkin and DJ-1 may be operative in the pathway of PINK1/parkin-mediated mitophagy.

Pathogenic LRRK2-mediated autophagy has been observed in a variety of cell cultures [149,150], in neurons derived from patient-induced pluripotent stem cells [151] and in animal models in which a mutant form of LRRK2 is expressed [152]. Either knock-down of LRRK2 by siRNA or treatment with a LRRK2 kinase inhibitor caused an increase in autophagic fluxin-cultured cells [153,154]. Overexpression of LRRK2, especially mutant forms, seems to suppress autophagy [155]. In contrast, studies from other two groups show that mutant forms of LRRK2 may induce autophagy via an ERK1/2-dependent pathway [149,156]. Although the detailed mechanisms by which LRRK2 and its mutants mediate autophagy are not clear, LRRK2 may disrupt the balance of autophagy through damaging lysosome-related calcium storage and cargo degradation [157,158]. Further, our group recently reported excessive mitophagy in a variety of cells expressing the LRRK2 G2019S mutant, which was accompanied by mitochondrial depolarization, recruitment of p62 to the mitochondria, increased LC3II levels and lysosomal activity as well as death of dopaminergic neurons [123,159]. We showed that the LRRK2 G2019S mutant caused excessive mitophagyby phosphorylating its substrate including fission protein Drp1 and mitochondrial outer membrane protein Bcl_2_ [123,159]. Thus, the pathway that LRRK2 mediation of mitophagy might be different from those occurred during autophagy.

Taken together, a large body of studies indicates that the different PD-related genes contribute to the pathogenesis of PD at the intersection of mitochondrial dysfunction and autophagy. The loss of mitochondrial membrane potential, which is associated with mitochondrial dysfunction, seems to be a common signal for mitochondria to be degraded via mitophagy. Moreover, occurrence of mitochondrial fragmentation due to impairment of mitochondrial fusion and fission often precedes the induction of mitophagy. Thus, it is possible that mitophagy may be a multistep process starting with the degradation of profusion/fission proteins, resulting in an imbalance of mitochondrial dynamics and the subsequent clearance of mitochondria [86]. However, the factors that regulate these processes involved in mitochondrial “quality control”, including mitochondrial dynamics, mitochondria-associated degradation and mitophagy in PD, remain to be determined.

## 6. Mitochondrial Redox Signaling in Parkinson’s Disease

Decreased Complex I activity in the SN of PD patients and animal models has been repeatedly observed. The defect in complex I results in impairment of electron transport and causes ROS accumulation in mitochondria which lead to neuronal degeneration. Neurotoxins causing parkinsonism, MPP^+^ are selectively taken up into dopaminergic neurons in which it inhibits Complex I activity [160]. Rotenone also inhibits Complex I by impairing oxidative phosphorylation [161]. These studies demonstrate a contribution from ROS to the pathogenesis of dopamine neuronal loss in PD. The genetic PD-linked proteins play a significant role in this process. Alpha-synuclein mutant A53T can enter mitochondria where it binds to the Complex I subunit to inhibit Complex I activity, producing ROS [162,163]. Functional studies showed that alpha-synuclein associated with mitochondria induces cytochrome c release, increased calcium and ROS levels resulting in dopaminergic neuronal death [164]. In addition, expression of mutants of PD-related genes Parkin, PINK1, DJ-1 and LRRK2 in cultured cells all increased ROS. This evidence has been well-summarized in other reviews [29,165]. However, it is uncertain if such elevated ROS are directly caused by these mutants or through indirect cellular effects.

Besides ROS, reactive nitrogen species (RNS) mediating nitrosative stress is also implicated in SN neuronal loss in PD [166]. RNS are generated by the reaction of superoxide with nitric oxide (NO), which results in the production of peroxynitrite. NO inhibits several enzymes including complexes I and IV of the mitochondrial electron transport chain, which in turn lead to ROS generation [167,168]. Increased expression of iNOS and nNOS were observed in basal ganglia of postmortem brain of PD patients [169]. In the mouse MPTP model, there was a significant upregulation of iNOS associated with the gliosis in the SN [170]. Inhibition of nNOS has been reported to protect against neurotoxicity in MPTP-induced PD animal model [171,172]. These observations suggest that NO and its metabolite peroxynitriteare implicated in the pathogenesis of PD.

## 7. Mitochondrial Protein Import in Parkinson’s Disease

Mitochondria possess their own DNA and translational machinery; however, there are only a small number of mitochondrial proteins encoded by mtDNA that are synthesized within the organelle. The majority of mitochondrial proteins are nuclear-encoded and have to be imported into the organelle. The translocase of the outer mitochondrial membrane (TOM complex) plays central roles in controlling protein entry to mitochondria [173]. The TOM complex is the main entry portal for most mitochondrial proteins that are synthesized in cytoplasm. The TOM complex contains seven subunits including TOM40, TOM22, two proprotein receptors, TOM20, and TOM70, and three smaller proteins, TOM5, TOM6 and TOM7. In general, imported proteins bind to one of these receptors. With the assistance of TOM22 and TOM5, the imported proteins pass the TOM 40 channel [174]. TOM complexes also need to cooperate with TIM (Translocase of the Inner Membrane) 23 complexes to import matrix-targeted proteins [175]. Thus, the TOM and the TIM23 complex direct the translocation of oxidative phosphorylation and metabolite transporter proteins to the inner membrane.

A number of studies have reported that PD-associated genes, alpha-synuclein, PINK1 and Parkin can interact with the TOM complex, disrupting the mitochondrial protein import. Alpha-synuclein can enter mitochondria where it mainly localizes at the inner membrane. The import of alpha-synuclein to mitochondria is through the TOM complex [176,177]. In postmortem brain samples of PD and in brain tissues of mice overexpressing alpha-synuclein, TOM 40 levels changed together with the levels of alpha-synuclein [177]. Expression with either wild-type alpha-synuclein or mutant A53T in cultured cells resulted in the loss of TOM40, whereas over-expression of TOM40 prevented the cellular damage caused by expression of either alpha-synuclein wild-type or its mutant A53T [177]. Thus, alpha-synuclein mutants may impair mitochondrial function by suppression of TOM40-dependent mitochondrial protein import pathways. PINK1 is also imported into the mitochondria via the TOM complex channel [178,179] and then is degraded in a membrane potential-dependent manner. Under mitochondrial depolarization PINK1, interacting with the TOM complex, recruits and activates Parkin, leading to degradation of mitochondrial outer membrane proteins and ultimately mitophagy [178]. Thus, pathogenic mutations in PINK1 and parkin may disrupt this pathway, resulting in the accumulation of dysfunctional mitochondria.

## 8. Potential Therapeutics Targeting Mitochondria for Treatment of PD

Given the importance of mitochondrial dysfunction in the pathogenesis of PD, therapeutics targeting mitochondria have been studied to prevent or treat PD. Although the cellular and pathological phenotypes from neurotoxin-induced and genetic mutant-associated PD models are different, the outcomes of mitochondrial dysfunction, including mitochondrial dynamic impairments, increased ROS, and impaired bioenergetics, seem to be common pathways. Thus, the mechanistic information on mitochondrial dysfunction in PD models provides potential targets for the development of therapeutic approaches for treatment of PD.

Here, we summarize reported therapeutic agents that reduce PD pathology in models of PD (see Table 2). We categorize these agents into (1) modulating PD-related genetic mutants; (2) modulating mitochondrial proteins; and (3) modulating the consequences of mitochondrial dysfunction. In Table 2, we list the neuroprotective effects of these agents especially in either animal models of PD or neuron derived patient-iPS cells.

*Modulating PD-related genetic mutants*: LRRK2 mutants are the most common genetic mutants in both familial and sporadic PD. Modulation of the LRRK2 kinase domain has been attractive for the development of therapeutics of PD. LRRK2 inhibitors, PF-06447475 and GW5074, have been shown to increase dopaminergic neuronal survival in primary neuronal cultures and neurons- derived from patient iPS cells [180,181]. Increasing glyoxalase activity of DJ-1 by supplying D-lactate and glycolate rescues the requirement for DJ-1 in maintenance of mitochondrial potential, increases cytocatalytic rate of DJ-1, and reduces neuronal death caused by paraquat and down-regulation of PINK1 [182]. However, thus far, these reagents are only tested in cultured cells so far. Whether treatment with these pharmacological agents protect neurons from PD in animal models remains to be determined.

**Table 2:** Therapeutic agents that target mitochondria for treatment of Parkinson’s disease

Category	Agent	Molecular Action	PD Mode	Therapeutic Effects
Modulating PD-related genes	PF-06447475	LRRK2 kinase inhibitor	Transgenic rat with LRRK2 G2019S	Reduce behavioral and neuropathological phenotypes
				Reduce inflammation [183]
	GW5074	LRRK2 kinase inhibitor	DA neurons derived from PD patient iPS cells	Suppression of ROS
				Improve mitochondrial respiration
				Increase DA neuronal survival [180]
	FX2149	LRRK2 GTPase inhibitor	mouse inflammation model	Reduce neuroinflammation
				Inhibit microglial activity [184]
Modulating mitochondrial protein	Alda-1	ALDH2 activator	Rotenone- and MPTP-induced animal models	Improve mitochondrial membrane potential
				Inhibit mitochondrial ROS
				Reduce dopaminergic cell death [185]
	TRO40303	inhibitor of mitochondrial transition pore	Mice expressing alha-synuclein	Upregulate mitochondrial proteins
				Increase TH expression [186]
	P110	Peptide inhibitor of Drp1	DA neurons from LRRK2 G2019S PD patient iPS cells	Improve mitochondrial membrane potential
				Inhibit mitochondrial ROS
				Increase mitochondrial integrity
				Reduce autophagy
				Improve DA neuronal morphology and survival [123]
	Mdivi-1	Inhibitor of mitochondrial fragmentation	MPTP-induced mouse PD model	Improve mitochondrial morphology
				Improve mouse behavioral outcome
			PINK1^−/−^ mouse model	Reduce DA neuronal loss in SN
				Restore dopamine level [187]
Modulating mitochondrial dysfunction	Q1	8-OH-quinoline-based iron chelator	MPTP-induced mouse PD model	Reduce DA neuronal degeneration in SN
				Decrease mitochondrial iron pool [188]
	Rapamycin	mTOR inhibitor	6-OHDA-induced rat PD model	Inhibit oxidative stress
				Inhibit mitochondrial apoptosis [189]
	Edaravone	ROS scavenger	Rotenone-induced rat PD model	Inhibit mitochondrial apoptosis
				Reduce ROS [190]
	Melatonin	Antioxidant	Rotenone-induced rat PD model	Suppress calcium level
			6-OHDA-induced rat PD model	Inhibit mitochondrial ROS
				Enhance complex I activity [191,192]
	Quercetin	Bioflavonoid	Rotenone-induced rat PD model	Inhibit mitochondrial ROS generation
				Inhibit p53 level
				Inhibit nuclear translocation of NF-kappaB
				Inhibit mitochondrial apoptosis [193]
	CNB-001	Curcumin derivative	MPTP-induced mouse PD model	Improve mitochondrial morphology
				Inhibit mitochondrial apoptotic pathway
				Improve mitochondrial membrane potential [194]
	Alpha-Lipoic acid	Antioxidant	Rotenone-induced rat PD model	Increase mitochondrial complex I activity
				Inhibit ROS generation
				Increase mitochondrial biogenesis
				Increase glutathione [195]
	Lycopene	Chemical carotene	Rotenone-induced rat PD model	Inhibit mitochondrial apoptotic pathway
				Increase SOD activity
				Increase glutathione
				Inhibit lipid peroxidation [196]

*Modulating mitochondrial proteins*: Aldehyde dehydrogenase 2 (ALDH2), located in mitochondrial matrix, functions as a cellular protector against oxidative stress by detoxification of cytotoxic aldehydes. Alda-1 is a small molecule that enhances ALDH enzyme activity and protects against oxidative toxicity [197]. Treatment with Alda-1 can reduce rotenone-induced apoptosis in both SH-SY5Y cells and primary dopaminergic neurons. Moreover, intraperitoneal administration of Alda-1 can improve mitochondrial membrane potential, inhibit mitochondrial ROS and reduce death of tyrosine hydroxylase (TH)-positive dopaminergic neurons in rotenone- or MPTP-induced PD animal models [185]. Cholesterol oximes such as olesoxime and TRO40303 are small molecules that interact with the mitochondrial outer membrane protein VDAC and limit opening of the mitochondrial transition pore in response to oxidative stress [198]. Olesoxime can protect differentiated SHSY-5Y cells from cell death, and reduce neurite retraction and cytoplasmic shrinkage induced by alpha-synuclein overexpression [199]. Low dose TRO40303 upregulates a number of mitochondrial proteins including Drp1 and VDAC and enhances expression of tyrosine hydroxylase in mice overexpressing alpha-synuclein [186]. As mentioned above, our group has developed a peptide inhibitor P110 that selectively blocks the protein-protein interactions between Drp1 and its mitochondrial adaptor Fis1 [125]. Treatment with P110 significantly reduced mitochondrial fragmentation, decreased mitochondrial ROS and improved mitochondrial integrity in dopaminergic neurons exposed to MPP^+^ in neurons expressing the LRRK2 G2019S mutation, and in dopaminergic neurons derived from LRRK2 G2019S patient-iPS cells [123,125]. Importantly, we showed that treatment with P110 had minor effects on mitochondrial dynamics and neuronal survival under physiological conditions [123,125]. In addition, Mdivi-1, an inhibitor of mitochondrial fragmentation, was reported to reduce behavioral and neuropathological phenotypes in an MPTP-induced PD mouse model, in addition to its protective effects in dopaminergic neurons exposed to neurotoxins [187].

*Modulating the consequences of mitochondrial dysfunction*: Modulation of downstream mitochondrial dysfunction may also provide therapeutic opportunities, in both sporadic and familial PD. In the past decades, a number of natural products and small molecules have been reported to protect against neuropathology associated with PD, at least in part through protecting mitochondria. Treatment with these agents has been shown to reduce mitochondrial ROS, inhibit mitochondrial apoptotic pathways, and increase mitochondrial complex I activity. As a consequence, treatment with these agents can reduce neuronal degeneration in PD in culture and in animals. These agents have been extensively reviewed [200,201,202,203]. Here, we only list those that have significant protection in animal models of PD (Table 2).

## 9. Concluding Remarks

Accumulating evidence supports the hypothesis that mitochondrial abnormalities and dysfunction could critically influence neuronal degeneration in both sporadic and faimilial PD. Dysregulation of mitochondrial dynamics and mitophagy have been centrally implicated in the neuropathology of PD. This evidence supports the idea that mitochondrial damage might be a primary cause initiating the progression of PD. Thus, targeting mitochondria may offer the opportunities for drug development to treat neurodegenerative diseases such as parkinsonism.
